# Sensitivity, specificity and predictive values of linear and nonlinear indices of heart rate variability in stable angina patients

**DOI:** 10.1186/1755-7682-5-31

**Published:** 2012-10-30

**Authors:** Flávio Correa Pivatelli, Marcio Antonio dos Santos, Gislaine Buzzini Fernandes, Marcio Gatti, Luiz Carlos de Abreu, Vitor E Valenti, Luiz Carlos M Vanderlei, Celso Ferreira, Fernando Adami, Tatiana Dias de Carvalho, Carlos Bandeira de Mello Monteiro, Moacir Fernandes de Godoy

**Affiliations:** 1 Serviço de Hemodinâmica e Cardiologia Intervencionista do Hospital de Base – Fundação Faculdade Regional de Medicina (FUNFARME), de São José do Rio Preto (SP) and Departamento de Cardiologia e Cirurgia Cardiovascular da Faculdade de Medicina de São José do Rio Preto (FAMERP), Av. Brigadeiro Faria Lima, 5416, 15090-000, São José do Rio Preto, SP, Brazil; 2 Departamento de Enfermagem em Saúde Coletiva e Orientação Profissional (DESCOP) do Curso de Enfermagem da Faculdade de Medicina de São José do Rio Preto (FAMERP), Av. Brigadeiro Faria Lima, 5416, 15090-000, São José do Rio Preto, SP, Brazil; 3 Departamento de Especialidades Cirúrgicas da Faculdade de Medicina de São José do Rio Preto (FAMERP), Av. Brigadeiro Faria Lima, 5416, 15090-000, São José do Rio Preto, SP, Brazil; 4 Laboratório de Escrita Científica, Departamento de Morfologia e Fisiologia, Faculdade de Medicina do ABC, Av. Príncipe de Gales, 821, CEP 09060-650, Santo André, SP, Brazil; 5 Programa de Pós-Graduação em Fisioterapia, Faculdade de Ciências e Tecnologia, Universidade Estadual Paulista, UNESP, Rua Roberto Simonsen, 305, 19060-900, Presidente Prudente, SP, Brazil; 6 NUTECC – Núcleo Transdisciplinar para Estudo do Caos e da Complexidade (FAMERP), Av. Brigadeiro Faria Lima, 5416, 15090-000, São José do Rio Preto, SP, Brazil; 7 Rua Garabed Karabashian 570, 15070-600, São José do Rio Preto, SP, Brazil

**Keywords:** Heart rate variability, Nonlinear dynamics, Coronary artery disease, Cardiovascular physiology, Stable angina

## Abstract

**Background:**

Decreased heart rate variability (HRV) is related to higher morbidity and mortality. In this study we evaluated the linear and nonlinear indices of the HRV in stable angina patients submitted to coronary angiography.

**Methods:**

We studied 77 unselected patients for elective coronary angiography, which were divided into two groups: coronary artery disease (CAD) and non-CAD groups. For analysis of HRV indices, HRV was recorded beat by beat with the volunteers in the supine position for 40 minutes. We analyzed the linear indices in the time (SDNN [standard deviation of normal to normal], NN50 [total number of adjacent RR intervals with a difference of duration greater than 50ms] and RMSSD [root-mean square of differences]) and frequency domains ultra-low frequency (ULF) ≤ 0,003 Hz, very low frequency (VLF) 0,003 – 0,04 Hz, low frequency (LF) (0.04–0.15 Hz), and high frequency (HF) (0.15–0.40 Hz) as well as the ratio between LF and HF components (LF/HF). In relation to the nonlinear indices we evaluated SD1, SD2, SD1/SD2, approximate entropy (−ApEn), α1, α2, Lyapunov Exponent, Hurst Exponent, autocorrelation and dimension correlation. The definition of the cutoff point of the variables for predictive tests was obtained by the Receiver Operating Characteristic curve (ROC). The area under the ROC curve was calculated by the extended trapezoidal rule, assuming as relevant areas under the curve ≥ 0.650.

**Results:**

Coronary arterial disease patients presented reduced values of SDNN, RMSSD, NN50, HF, SD1, SD2 and -ApEn. HF ≤ 66 ms^2^, RMSSD ≤ 23.9 ms, ApEn ≤−0.296 and NN50 ≤ 16 presented the best discriminatory power for the presence of significant coronary obstruction.

**Conclusion:**

We suggest the use of Heart Rate Variability Analysis in linear and nonlinear domains, for prognostic purposes in patients with stable angina pectoris, in view of their overall impairment.

## Background

One method to evaluate the cardiac autonomic function is the analysis of heart rate variability (HRV) which is the variation in time between consecutive sequences of normal RR intervals, also called NN intervals [1-4]. This variation is controlled mainly by the autonomic nervous system (ANS) by the direct action of efferent vagal and sympathetic nerves on receptors located in the sinoatrial node. Other factors such as circadian rhythms, thermoregulation, humoral systems and respiratory sinus arrhythmia can also be related to HRV either directly or via SNA. Therefore the various physiological and pathological states that alter the autonomous activity may change the variability of RR intervals [1]. Nowadays, HRV analysis using nonlinear methods has been receiving increasing attention. There is evidence that mechanisms involved in cardiovascular regulation, likely interact between each other in a nonlinear fashion [5,6]. One method used for this purpose is Detrended Fluctuation Analysis (DFA), which quantifies the presence or absence of fractal correlation properties of the RR intervals [5]. According to Tulppo et al. [7], fractal indices are able to detect slight changes in the dynamics of RR intervals better than conventional spectral analyses. Moreover, impairment of fractal correlation properties of short- and long-term dynamics of HRV helps clinical professionals to detect autonomic dysfunction and avoid disease development.

Patients with reduced HRV, as assessed by nonlinear methods in the preoperative period of surgical myocardial revascularization, tend to have higher morbidity and mortality [1]. In addition, Correa and coworkers [8] indicated that the nonlinear dynamics methods, at their respective cut-off levels, allowed for the identification of patients developing pulmonary infection in the postoperative period of surgical myocardial revascularization. However, it is not clear in the literature if this method has a prognostic value in coronary arterial disease subjects. Therefore, we evaluated the sensitivity, specificity and predictive values of the linear and nonlinear indices of HRV in stable angina patients submitted to coronary angiography.

## Method

### Study population

We studied 77 unselected patients from the Catheterization Laboratory for elective coronary angiography because of a history of stable angina. All patients signed a consent letter and all procedures were approved by the Ethical Committee in Research (Protocol number 003/08).

### Exclusion criteria

We excluded patients with history of myocardial infarction, heart valve disease, congenital heart disease, coronary artery bypass grafting or percutaneous coronary intervention, pacemaker, cardiac transplantation and pregnancy. We did not exclude patients with classic risk factors for coronary artery disease (CAD). Patients with no significant obstruction, i.e. > 0% to <50% were excluded from analysis.

### Angiographic evaluation

Patients underwent coronary angiography and left ventriculography using the SHIMATZU 2400 or PHILIPS equipment. For eccentric lesions, the projection with the highest degree of stenosis was used. The quantification of lesions was performed visually as well as left ventricular function. The patients were divided into two groups depending on the severity of coronary arterial disease (CAD). Non-CAD Group was defined as patients without obstructions and CAD Group those with one or more obstructions >50%. Left ventricular function was classified as no significant dysfunction (0) or with significant dysfunction.

### Heart rate variability analysis

The heart monitor strap was placed on each subject’s thorax over the distal third of the sternum. The heart rate (HR) receiver (Polar S810i monitor, Polar Electro OY, Kempele, Finland) was placed on the wrist. This equipment has been previously validated for beat-by-beat measurements and for HRV analysis [9,10]. The subjects were placed in the dorsal decubitus position on a cushion and remained at rest with spontaneous breathing for 40 minutes. After editing the data we selected the first 1000 RR intervals of each recording for analysis.

### Linear Indices of HRV

Analysis in the time domain was performed by means of SDNN (standard deviation of normal-to-normal RR intervals), RMSSD (root-mean square of differences between adjacent normal RR intervals in a time interval) and total number of adjacent RR intervals with a difference of duration greater than 50ms (NN50). To analyze HRV in the frequency domain, we evaluated the following components: ultra-low frequency (ULF) ≤ 0.003 Hz, very low frequency (VLF) 0.003 – 0.04 Hz, low frequency (LF) (0.04–0.15 Hz), and high frequency (HF) (0.15–0.40 Hz) as well as the ratio between LF and HF components (LF/HF). The spectral analysis was calculated using the Fast Fourier Transform algorithm. For analysis of linear indexes in the time and frequency domains, we used the HRV analysis software from Kuopio University [10].

### Nonlinear Indices of HRV

For non-linear analysis were used the following variables: DFA (Detrended Fluctuation Analysis with the components α1 and α2), Approximate Entropy (ApEn-), Hurst exponent (HE) and the Lyapunov exponent (LE) [9]. For this purpose we used the softwares DFA and Chaos Data Analyzer Version 2.1. The nonlinear geometric variables SD1 and SD2 were obtained by the Poincaré plot using the software HRV Analysis Software [10].

### Statistical analysis

Data were analyzed by using the StatsDirect Statistical Software program, version 2.5.7. The univariate comparison of demographic data between groups (with CAD and without CAD) was performed with the chi-square test for nominal variables and the unpaired t test for continuous variables. We used the nonparametric Mann–Whitney test to evaluate continuous variables that did not present a Gaussian distribution. The definition of the cutoff point of the variables for predictive tests was obtained by the Receiver Operating Characteristic curve (ROC). The area under the ROC curve was calculated by the extended trapezoidal rule, assuming as relevant areas under the curve ≥ 0.650. All tests showed that the area under the ROC curve ≥ 0.650 were used in logistic regression analysis adjusted for age.

## Results

Among all patients, 13 were excluded because they did not present significant obstruction (> 0% and <50%). Among the 64 remaining patients, 21 (32%) belonged to CAD Group and 43 (67%) to non-CAD Group. The demographic and clinical data are listed in Table 1. The groups were not similar only in age (49 ± 9 vs 61 ± 10, p <0.0001). Regarding the extent of coronary disease in the CAD group, 16 patients had uniarterial and 27 multi-arterial disease.

**Table 1:** Demographic and clinical data

**Variables**	**No DAC (n = 21)**	**DAC (n=43)**	**p**
Age (years)	50±10	62±10	< 0.0001
Male gender	42.9%	69.8%	0.0563
BMI (kg/m^2^)	28±6	28±5	0.7119
Relevant LV dysfunction	0%	33%	0.0854
Hypertension	23.5%	62.8%	0.1142
Hypercholesterolemia	14.7%	34.9%	0.4074
β Blockers	17.7%	48.8%	0.1785
Ca channels blockers	2.9%	16.3%	0.2546
ACE inhibitor	17.7%	51.2%	0.1114
Statins	11.8%	23.3%	1

BMI: Body mass índex; ACE: Angiotensin converting enzyme.

The indices SDNN, RMSSD, NN50 and HF were significantly reduced in patients with obstructive coronary disease. Regarding the non-linear measures, only the geometric variables SD1, SD2 and approximate entropy indicated a significant difference (Table 2).

**Table 2:** HRV indices in no CAD and CAD groups

**Index**	**No DAC (n = 21)**	**DAC (n=43)**	**p**
**RR (ms)**	896±136	914±139	0.6191
**SDNN (ms)**	39.71±18.7	29.95±13.6	0.0209 *
**RMSSD (ms)**	32.38±18.1	22.99±11.9	0.0303 **
**NN50 (count)**	135.48±146.28	59.42±80.9	0.0476 **
**VLF (ms**^**2**^**)**	323.67±420.49	220.56±242.8	0.2058
**LF (ms**^**2**^**)**	332.48±420.78	179.12±175.08	0.0641
**HF (ms**^**2**^**)**	233.10±241.7	87.72±81.04	0.0073 **
**SD1 (ms)**	23.21±12.9	16.52±16.52	0.0282 *
**SD2 (ms)**	67.19±25.9	52.75±25.6	0.0387 *
**SD1/SD2**	0.33±0.1	0.34±0.1	0.6269
**α1**	0.93±0.2	1±0.3	0.2189
**α2**	0.9±0.2	0.91±0.1	0.5577
**Lyapunov exponent (LE)**	0.91±0.1	0.89±0.1	0.4416
**Hurst exponent (HE)**	0.21±0.1	0.22±0.1	0.6294
**-ApEn**	0.388±0.09	0.343±0.08	0.0368 **

* = p <0.05 with area under ROC curve < 0.65; ** = p <0.05 with area under ROC curve ≥ 0.65.

Table 3 shows the sensitivity, specificity, predictive value, area under the ROC curve and ODDS Ratios with 95% confidence interval (95%) of all variables. The variables that presented the best discriminatory power (area under the ROC curve ≥ 0.650) for the presence of significant coronary obstruction were HF, RMSSD, NN50 and –ApEn. When we adjusted it for age excluding those aged more extreme, i.e., those with <40 or> 62 years old, the variable -ApEn and HF remained with significant discriminatory power, with a slight drop for RMSSD and NN50 (Table 4).

**Table 3:** Sensitivity, Specificity, and Predictive Value of ROC area of the HRV indices to assess the presence of CAD

**Variable/Cutoff**	**Sens**	**Sp**	**PPV**	**NPV**	**Area under ROC curve**	**p**	**Odds Ratio** **(IC 95%)**
**HF ≤ 65 (ms**^**2**^**)**	58%	81%	49%	39%	0.708	0.0037	5.90 (1.7-20.53)
**RMSSD ≤ 24.55 (ms)**	70%	67%	81%	52%	0.667	0.0076	4.62 (1.51-14.1)
**NN50 ≤ 15.54 (count)**	51%	81%	85%	45%	0.653	0.0164	4.45 (1.16-20.7)
**-ApEn ≤ 0.296**	40%	90%	89%	42%	0.666	0.0188	6.21 (1.28-30.17)

Sens = sensitivity, Sp = specificity, PPV = positive predictive value, NPV = negative predictive value.

**Table 4:** Sensitivity, Specificity, and Predictive Value of ROC area of the HRV indices to assess the presence of CAD according to the patients’ age

**Variable/Cutoff**	**Sens**	**Sp**	**PPV**	**NPV**	**Area under ROC curve**
**-ApEn ≤ 0.41**	87%	47%	69%	73%	0.698
**HF ≤ 198.19 (ms**^**2**^**)**	96%	41%	69%	88%	0.669
**RMSSD ≤ 19.53 (ms)**	61%	71%	74%	57%	0.642
**NN50 ≤ 176.40 (count)**	91%	41%	68%	78%	0.625

Sens = sensitivity, Sp = specificity, PPV = positive predictive value, NPV = negative predictive value.

Table 5 shows the correlation between age and linear and nonlinear indices of HRV. There was no high correlation between these measures and age considering the total group. This suggests that we could maintain the four variables mentioned above as relevant in differentiating between patients with and without significant CAD.

**Table 5:** **Correlation (R**^**2**^**) between linear and nonlinear variables with age**

**Variable**	**R**^**2**^
**SDNN (ms)**	0.31
**RMSSD (ms)**	0.24
**VLF (ms**^**2**^**)**	−0.11
**LF (ms**^**2**^**)**	−0.39
**HF (ms**^**2**^**)**	−0.32
**α1**	<0.01
**α2**	0.22
**-ApEn**	<0.01

## Discussion

In this study we aimed to investigated linear and nonlinear indices if HRV in stable angina patients. We also evaluated the sensitivity, specificity and predictive values of those components. As a main finding, we reported that the indices which presented the best discriminatory power for the presence of significant coronary obstruction were HF ≤ 66 ms^2^, RMSSD ≤ 23.9 ms, ApEn ≤−0.296 and NN50 ≤ 16. Based on the linear indices evaluated in our study, parasympathetic activity was reduced in CAD patients. Previous studies support our findings, which is associated with changes in autonomic regulation on heart [11,12], in patients showing the symptoms of CAD the symptoms of CAD [13-15], and there is evidence that the reduction of heart rate variability as a result of this change may be related to the severity of hemodynamically significant coronary stenosis as well as progression of coronary atherosclerosis in humans [16,17]. Hayano et al. [16] demonstrated the existence of an association between reduction of vagal stimulation on the heart, using the analysis of spectral components, and angiographic severity of CAD. Although there is no consensus on the association between CAD severity and the reduction of HR variability, the most prominent changes detected by spectral measures is the reduction of the high frequency component (related to parasympathetic) in patients with uncomplicated CAD [12,18].

We reported that among the linear indices of HRV, RMSSD, NN50 and HF were those which presented the highest discriminatory power of the presence of CAD. These indices represent the heart rate fluctuations of short period, which are under the influence of the parasympathetic system [19,20]. These data suggest that discrimination of patients with CAD is related to the change in the modulation of vagal activity of the heart. Similar findings were obtained by Airaksinem et al. [12], which demonstrated the depression of vagal activity in CAD patients. In this study the HF index was the only variable in the frequency domain significantly different between the CAD subjects and those without CAD. The group composed of more severe disease presented the lowest values, which confirms the findings of the literature, showing reduction of vagal stimulation.

Wennereblom et al. [21] also found a reduction in the RMSSD index (29 vs 23, p=0.01) and pNN50 (8.2 vs 4.8, p=0.01) in patients with uncomplicated CAD. Taken together, our findings and Wennereblom et al. study [21] support the loss of HRV in patients with CAD.

Regarding the nonlinear indices, our study reported differences only in the approximate entropy. The approximate entropy is a nonlinear index that quantifies the regularity of a time series and the logarithmic likelihood that patterns of time series remain similar to each other following new additional comparison [22,23]. The higher the value of the approximate entropy, the greater the unpredictability in series, i.e., regular sequences will result in the lower approximate entropy, while the random behavior (and also the chaotic) is associated with high values of approximate entropy.

According to our study, the approximate entropy was the nonlinear index with the best discriminatory power of CAD. In the present study an individual with approximate entropy lower than or equal to 0.296 (for a series of 1000 beats) showed an increased likelihood of significant coronary obstruction, indicating that the CAD is a loss of complexity according to a lower HRV. In healthy adults the approximate entropy value is above 0.91 for 24 hour Holter analysis. The result of this study is consistent with that of Nikolopoulos et al. [24], which reported reduction of the approximate entropy in patients with uncomplicated CAD compared with healthy control subjects (0.24 ± 0.09 versus 1.22 ± 0.15). Other heart diseases present changes in the complexity of RR intervals, assessed by approximate entropy. Vikman et al. [25] revealed reduced approximate entropy preceding spontaneous onset of paroxysmal atrial fibrillation in patients without structural heart disease (0.89 ± 0.27 versus 1.02 ± 0.30).

Furthermore, we found no correlation between approximate entropy and spectral measurements implying that the information provided by the approximate entropy is different from that of linear measurements. Although there are differences between the groups regarding age, this index presented no influence on HRV considering that when we eliminated the extreme ages (<40 years and> 62), the predictive values of HRV measures with greater clinical significance remain stable. Likewise, it was not observed correlation between age and approximate entropy (R^2^<0.01). These data can also be explained by the small difference between the ages of the two groups (50 years old x 61 years old). A study has demonstrated that when performed detailed analysis by age group, after 40 years there is stabilization in the decline of the fractal dimension and approximate entropy [25,26]. Pikkujämsä et al. [27] also found no correlation between age with approximate entropy and DFAα1 in an age range between 40 and 59 years old, although the relationship has been identified with SDNN and LF indices.

Almost all studies evaluating HRV in patients with CAD involves carefully selected populations which do not reflect the general population. The analysis of HRV in not selected patients with stable angina and its predictive value for the presence of significant coronary obstruction is poorly known, justifying the present study.

Some authors analyze HRV in the records of long intervals of 24 hours. Our study shows that even a record of short duration (± 15 to 20 minutes) involving a series of 1000 beats, is sufficient to analyze HRV in patients with suspected CAD, separating cases without injury from those with severe obstructions.

There is insufficient knowledge of the exact mechanism that causes the reduction of HRV. Although there is intense research in various aspects of HRV, new models and large prospective evaluations will be needed before the widespread clinical application of this technique becomes possible.

The present study has certain limitations that need to be taken into account. We should mention the influence of drug therapy and the fact that the study sample was heterogeneous in terms of risk factors. Although we consider that the analysis of the influence on HRV of each risk factor individually is very important, this has proved impossible in the clinical practice, because this group of patients usually present with associated diseases and/or risk factors.

## Conclusion

The indices that present the best discriminatory power for the presence of significant coronary obstruction were HF in absolute units, RMSSD, ApEn and NN50. Also, parasympathetic activity was reduced in CAD patients. The study of heart rate variability, by being a noninvasive, low-cost and risk-free method, can be clinically relevant in the diagnostic evaluation of the presence or absence of significant coronary artery disease in patients with stable angina.

## Abbreviations

HRV: Heart rate variability; ANS: Autonomic nervous system; DFA: Detrended fluctuation analysis; CAD: Coronary artery disease; HR: Heart rate; ULF: Ultra-low frequency; VLF: Very low frequency; LF: Low frequency; HF: High frequency; SNDD: Standard deviation of normal-to-normal RR intervals; RMSSD: Root-mean square of differences between adjacent normal RR intervals in a time interval; NN50: Total number of adjacent RR intervals with a difference of duration greater than 50ms; ApEn: Approximate entropy; LE: Lyapunov exponent; HE: Hurst exponent.

## Competing interests

The authors declare that they have no competing interests.

## Authors’ contributions

FCP, MAS, GBF, MG, LCA, VEV, LCMV, CF, FA, TDC, CBMM and MFG participated in the acquisition of data and revision of the manuscript. All authors conceived of the study, determined the design, performed the statistical analysis, interpreted the data and drafted the manuscript. All authors read and gave final approval for the version submitted for publication.
